# Development of Dorzolamide Loaded *6-O*-Carboxymethyl Chitosan Nanoparticles for Open Angle Glaucoma

**DOI:** 10.1155/2013/562727

**Published:** 2013-10-10

**Authors:** Ujwala Shinde, Mohammed Hadi Ahmed, Kavita Singh

**Affiliations:** Department of Pharmaceutics, Bombay College of Pharmacy, Kalina, Mumbai, Maharashtra 400098, India

## Abstract

Chitosan (CS) is a biodegradable, biocompatible, and mucoadhesive natural polymer soluble in acidic pH only and can be irritating to the eye. Objective of the study was to synthesize water soluble *6-O*-carboxymethyl (OCM-CS) derivative of CS, and to develop CS and OCM-CS nanoparticles (NPs) loaded with dorzolamide hydrochloride (DRZ). CS was reacted with monochloroacetic acid (MCA) for OCM-CS synthesis and was characterized by FT-IR, DSC, and ^13^C NMR. CS and OCM-CS NPs were prepared by ionic gelation method. Ocular irritation potential were evaluated and therapeutic efficacy was measured by reduction in intraocular pressure (IOP) in normotensive rabbits. Maximum yield was obtained when the ratio of water/isopropyl alcohol was 1/4 at 55°C. The FT-IR, DSC and ^13^C NMR confirmed the formation of an ether linkage between hydroxyl groups of CS and MCA. The particle size and zeta potential of optimised CSNPs was 250.3 ± 2.62 nm and +33.47 ± 0.723 mV, whereas those for OCM-CSNPs were 187.1 ± 2.72 nm and 30.87 ± 0.86 mV. The entrapment efficiency was significantly improved for OCM-CSNPs, compared to CSNPs. OCM-CSNPs had tailored drug release and improved bioavailability with reduction in pulse entry as compared to CSNPs. Hence, it can be concluded that DRZ loaded OCM-CSNPs would be better alternative option to available eye drops for glaucoma treatment.

## 1. Introduction

Open-angle glaucoma, the most common form of glaucoma, accounts for at least 90% of all glaucoma. It is caused by clogging of Schlemm's canal, develops slowly, and has a wide angle between iris and cornea. Its symptoms and damage are unnoticeable and it is a lifelong condition. The major risk factor for glaucoma is elevated IOP. Lowering IOP is currently the only proven method for reducing the risk of glaucomatous visual field loss and remains the primary goal of therapy [1]. 

DRZ is a carbonic anhydrase inhibitor (CAI) used in the treatment of glaucoma. Carbonic anhydrase (CA) is responsible for generation of bicarbonate anions secreted by the ciliary process into the posterior chamber of the eye. Inhibition of CA results in reduction of IOP. Orally administered CAIs, such as acetazolamide, are very effective ocular hypotensive agents but their oral administration results in systemic side effects including general malaise, depression, loss of appetite, fatigue, weight loss, gastrointestinal disturbances, parenthesis, and renal calculi [2]. Dorzolamide is reported to have 20 times higher potency than acetazolamide and is topically active [3]. When dorzolamide solution is instilled in ocular cul de sac, common side effects observed are bitter taste in mouth, blurred vision, redness, burning and stinging upon instillation of eye drops, dryness of eyes with sensitivity to sunlight, and tearing. These side effects could be due to exposure of concentrated solution of dorzolamide to eyes and would be more severe when eye drops are instilled frequently to achieve the desired pharmacological effects.

Targeting the drug to the appropriate site of action in the eye is usually one of the greatest challenges in drug delivery because of its anatomical and physiological defense mechanisms. Ocular drug delivery systems thus compel specified criteria according to the physiological structure of the eye [4]. Conventional ocular preparations have the disadvantage of extremely low bioavailability, short precorneal residence time owing to the tear turnover, and rapid nasolacrimal drainage of the instilled drug from the tear fluid. Typically, less than 5% of the topically applied drug penetrates the cornea and reaches intraocular tissues. Frequent instillations are often required to achieve the required therapeutic effect, and this leads to escalating inconvenience and adverse effects [5]. Modified ocular drug delivery systems like ocular inserts and *in-situ* gelling systems, though providing some advantages in terms of extended drug delivery, and could not overcome the problems of blurred vision, sticking of the eyelids, undesirable systemic absorption, and low patient acceptance [6]. 

Retention of a drug delivery system in front of the eye is thus desirable to circumvent the loss of an instilled drug. Thus, the goal in ocular therapeutics is to maintain an effective drug concentration at the site of action for an appropriate period, in order to achieve the expected pharmacological response [7]. The use of nanosystems/colloidal carriers is claimed to provide numerous advantages for ocular drug delivery systems, because of their ability to protect the encapsulated molecule while facilitating its transport to the different compartments of the eye. Furthermore, nanosystems can provide controlled drug delivery for extended periods of time. It offers an attractive benefit for the treatment of chronic ocular diseases like glaucoma. When the use of drug preparation by the patient is lifelong, this purportedly patient-friendly approach is of utmost significance [8]. These colloidal carriers may be applied in liquid form in the same manner as eye drop solutions. By interaction with the glycoproteins of the cornea and conjunctiva, they can form a precorneal depot, resulting in a prolonged release of the bound drug [9]. 

Ocular bioadhesion, specifically, refers to the capacity of certain polymers to adhere to the mucus coat covering the conjunctival and corneal surfaces of the eye by noncovalent bonds. The clearance time of bioadhesive polymeric systems is delayed as its dependence is shifted to mucus turnover rate rather than tear turnover rate. The importance of bioadhesive polymers lies in their ability to significantly improve the performance of controlled delivery systems by enhancing the means of optimum contact with the absorbing surface. This ultimately prolongs the residence of the ocular dosage form in the cul de sac, which reduces dosing frequency [10]. 

CS is a natural polysaccharide obtained from crustacean shells and is composed of 2-amino-2-deoxy-*β*-D-glucan combined with glycosidic linkages. The primary amine groups render special properties that make CS very useful in pharmaceutical applications. The nontoxicity, biodegradability, and biocompatibility make CS suitable for drug delivery [11]. The potential application of CS is hindered by its limited aqueous solubility. Thus, CS is chemically modified to improve the polymer process ability, solubility, antimicrobial activity, and the ability to interact with other substances [12]. Introducing a carboxymethyl group is the most advantageous method of increasing the solubility of chitosan at neutral and alkaline pH without affecting other important characteristics. OCM-CS is an amphiprotic ether derivative of chitosan, containing –COOH and –NH_2_ groups in the molecule, exhibit nontoxicity, biodegradability, biocompatibility, antibacterial, and antifungal activity, and has therefore received considerable attention in biomedical applications [13]. This overview enabled us to conclude that polymeric nanoparticles could serve as a best suited option in topical ocular delivery.

The present study was planned with objective to chemically modify chitosan and to develop nanoparticulate system from chitosan and modified chitosan capable of delivering the DRZ in a sustained manner. Thus frequent instillation of dorzolamide would be avoided which otherwise may induce toxic side effects and cellular damage at the ocular surface.

## 2. Materials and Methods

### 2.1. Chemicals and Animals

DRZ was received as a gift sample from FDC Ltd, (Mumbai, India). CS (degree of deacetylation 90% and MW 400.0 KD), sodium tripolyphosphate (TPP), agarose, and monochloroacetic acid (MCA) were purchased from Sangam Laboratories (Mumbai, India), Sigma-Aldrich (St. Louis, MO, USA), GE Biosciences (Pittsburgh, USA) and S.D. Fine Chem. Ltd (Mumbai, India), respectively. All other chemicals were of AR grade quality and used as received. Fertile White Leghorn chicken eggs were procured from Central Poultry Development Organization (Mumbai, India). Rabbits were supplied by Bombay Veterinary College (Mumbai, India).

### 2.2. Methods

#### 2.2.1. Synthesis of OCM-CS

OCM-CS was synthesized as per the previously reported method with some modification [14, 15]. Briefly, CS (10 g) and sodium hydroxide (12.5 g) were added to solvent (100 mL) in a round bottom flask to alkalize and swell at 35°C for 2 h. The solvent consisted of water and isopropyl alcohol. MCA (13 g) was dissolved in the solvent blend containing distilled water and isopropyl alcohol (IPA), added to the hydrated alkaline CS dropwise over a period of 30 min and then reacted for 4 h at 55°C. After completion of the reaction 2.5 M hydrochloric acid (HCl) was added to neutralize the reaction mass and the solvent was decanted. Ethyl alcohol (80%) was added to precipitate, desalt, and dewater the OCM-CS. The product was dried under vacuum at room temperature. The dried product was dissolved in distilled water and subjected to dialysis for 3 days, after which it was lyophilized. The OCM-CS yield was optimized by varying solvent ratios and temperature conditions (Tables 1 and 2).

#### 2.2.2. Characterization of OCM-CS

*Fourier Transformation-Infrared (FT-IR) Spectroscopy.* Potassium bromide (KBr) 50 mg was thoroughly mixed with 10 mg of OCM-CS CS to prepare KBr disks with electrically operated KBr Press Model HP-15. Jasco FTIR-5300 spectrophotometer (JASCO, MD, USA) was used to obtain IR spectra of the prepared disc of OCM-CS and CS. The scanning range was 4000–400 cm^−1^.

*Differential Scanning Calorimetry.* The Differential scanning calorimetry (DSC) thermograms were obtained using DSC 6220 (SII Nanotechnology, Northridge, CA, USA). Briefly, about 10 mg of sample was placed in aluminum sample pan and sealed. The samples were heated from 0°C to 500°C at a heating rate of 10°C/min using nitrogen as purge gas (20 mL/min). The DSC was earlier calibrated using standard Alumina.

^13^ *C NMR Spectroscopy.* The ^13^C NMR spectrum of OCM-CS was acquired at 80°C by using a Mercury Plus 300 MHz NMR spectrometer (Varian Medical Systems, Inc., CA, USA). For acquiring the ^13^C NMR spectra of OCM-CS, its solution was prepared in D_2_O at concentration of 10 mg/mL.

*Content of Free Amino Group.* The content of free amino group is defined as the average number of free nitrogen atom of each saccharide unit in an OCM-CS molecule. It was measured by potentiometric titration [16]. 

About 0.2 g of OCM-CS, was added into 25.0 mL of standard HCl (0.1 M) solution and stirred until the sample was completely dissolved. The solution was then potentiometrically titrated with 0.1 M standard NaOH solution. According to the volume of NaOH between the second and third pH leap, the content of amino group was calculated from the formula

(1) Content  of  free  amino =(V3−V2)×CNaOH×240.07WSample,

where *V*_2_ and *V*_3_ are the volumes (mL) of standard NaOH solution used in the second and third pH leap, respectively, *C*_NaOH_ is the concentration of standard NaOH solution, *W*_Sample_ represents the weight (g) of the sample, and 240.07 is the average molecular weight of each saccharide unit in an OCM-CS molecule.

*Degree of Substitution.* Degree of substitution (DS) is the number of carboxymethyl group that binds to the glucosamine (skeleton unit) in an OCM-CS molecule. The DS of OCM-CS was determined by dissolving OCM-CS (300 mg) in 0.1 M HCl (30 mL) and titrating with 0.1 M aqueous NaOH. The DS value was calculated as follows [17, 18]:

(2) $$\begin{array}{c} \text{DS}=\frac{161A}{\left(M_{\text{OCM-CS}}-58A\right)},\\ A=V_{\text{NaOH}}\cdot C_{\text{NaOH}}, \end{array}$$

where *V*_NaOH_ and *C*_NaOH_ were the volume and molarity of aqueous NaOH, respectively, *M*_OCM-CS_ was the mass of OCM-CS, and 161 and 58 are the molecular weights of the glucosamine and the carboxymethyl group, respectively.

#### 2.2.3. Preparation of OCM-CS and CS NPs

In the present study, OCM-CS and CS NPs were prepared by ionic gelation method which was induced by adding 1% w/v solutions of calcium chloride (CaCl_2_) and TPP, respectively [19, 20]. Briefly, OCM-CS (4 mg/mL) was dissolved in distilled water whereas CS (4 mg/mL) was dissolved in glacial acetic acid and pH of CS solution was adjusted to 5.5 with sodium acetate buffer pH 5.5. OCM-CS and CS NPs were formed spontaneously at room temperature when CaCl_2_ and TPP solutions were added to the aqueous solution of OCM-CS and CS under magnetic stirring, respectively. After complete addition of CaCl_2_ and TPP, solutions were further stirred for 30 min to ensure complete gelation of NPs. Different weight ratios (1 : 1 to 6 : 1) of OCM-CS : CaCl_2_ and CS : TPP were used in order to determine the weight ratio providing NPs with optimum nanosize particles. For the preparation of drug-loaded NPs, DRZ was added in the polymer solution. Different DRZ concentrations were used, 10%, 20%, 25%, 50%, and 75% (w/w) based on polymer weight, in order to study the effect of the DRZ loading on the particle size, PI, zeta potential, entrapment efficiency, and *in vitro* release profile. Every batch was prepared in triplicate and the results represent the average value. The DRZ loaded NPs were centrifuged at 30,000 rpm for 30 min at 4°C using Optima Max XP ultracentrifuge (Beckman Coulter, USA), supernatant was discarded, and pellets were resuspended in distilled and lyophilized by adding mannitol as cryoprotectant.

*Particle Size PI and Zeta Potential of NPs.* The particle size distribution, NPs was determined by dynamic light scattering (DLS) using a Malvern Zetasizer Nano ZS (Malvern Instruments Ltd., Worcestershire, UK) at 25°C. The NP dispersions were diluted in distilled and filtered water (Millex-HV Filter, 0.45 *μ*m, Millipore, Billerica, MA, USA) up to a count rate of 50 to 300 Kcps (1000 counts per second). The data reported were particle size, evaluated as the intensity obtained from three repeat measurements, and the polydispersity index [20]. Before measurement of zeta potential, nanoparticulate dispersions were diluted with filtered 1 mM NaCl solution (Millex-HV Filter, 0.45 *μ*m, Millipore, Billerica, MA, USA) up to a count rate of 100 to 1000 Kcps. All measurements were performed in triplicate.

*Evaluation of Entrapment Efficiency of NPs.* Entrapment efficiency of DRZ loaded NPs was determined according to the previously reported method [20]. For the determination of the entrapment efficiency, the NPs were first separated from the aqueous suspension medium by ultracentrifugation at 40,000 rpm for 30 min using Optima Max XP ultracentrifuge (Beckman Coulter, USA). The entrapment efficiency was determined in triplicate indirectly by analyzing the amount of free DRZ in supernatant. The free DRZ in supernatant was quantified by validated UV spectrophotometric method at 254 nm. The entrapment efficiency of DRZ NPs was calculated as follows:

(3) $$\begin{array}{c} \text{Entrapment}\;\text{efficiency}=\frac{\left[\text{Total}\;\text{amount}\;\text{of}\;\text{DRZ}\;\text{loaded}-\text{Free}\;\text{DRZ}\;\text{in}\;\text{supernatant}\right]}{\text{Total}\;\text{amount}\;\text{of}\;\text{DRZ}\;\text{loaded}\;}\times 100. \end{array}$$

*In Vitro Drug Release of NPs.* The *in vitro* drug release profiles of optimized, lyophilized, and sterilized DRZ loaded NPs were determined in 50 mL simulated tear fluid, pH 7.4 (STF) using dialysis bags (Himedia Laboratories, India) at 37°C under magnetic stirring. At predetermined time intervals, 5 mL aliquots were withdrawn from the medium and analyzed for DRZ by validated UV spectrometry. To determine the release behavior of free drug, *in vitro* study of DRZ solution (2% w/v) was also performed as control experiment [20]. Studies were performed in triplicates and data was analyzed for release kinetics.

*In Vitro Mucoadhesion of NPs*. The binding efficiency of mucin to NPs was determined by mixing 2 mL of mucin (625 *μ*g/mL) with the same volume of NPs. NPs were previously ultra centrifuged at 40,000 rpm for 30 min and resuspended in distilled water. After incubation at 37°C for 30 min, the samples were ultracentrifuged at 40,000 rpm for 30 min. The concentration of free mucin in the supernatant was determined at 555 nm by periodic acid/Schiff (PAS) colorimetric method [21, 22]. The mucin binding efficiency of NPs was calculated from the following equation:

(4) $$\begin{array}{c} \text{Mucin}\;\text{binding}\;\text{efficiency}\;\left(\%\right)=\left[\frac{\left(C_{0}-C_{s}\right)}{C_{0}}\right]\times 100, \end{array}$$

where *C*_0_ is the initial concentration of mucin used for incubation and *C*_*s*_ is the concentration of free mucin in the supernatant.

*FT-IR Spectroscopy and DSC Analysis of NPs.* IR spectra and DSC thermograms of lyophilized OCM-CS and CS NPs (without cryoprotectant) were obtained to study any possible interaction and to characterize the thermal behavior between polymers and DRZ.

*Morphological Characterization of NPs.* Transmission electron microscopy (TEM) was used to examine the morphology of the OCM-CSNs. TEM micrographs were obtained with Philips CM-200 (PHILIPS, The Netherlands). The sample of OCM-CSNs (5–10 *μ*L) was dropped onto Formvar coated copper grids. After complete drying, the sample was stained using 2% (w/v) phosphotungstic acid. Digital Micrograph and Soft Imaging Viewer software were used to perform the image capture and analysis, including particle sizing. 

*Ocular Irritation Potential Test.* The hen's egg test on the chorioallantoic membrane (HET-CAM) is the alternative method to animal experimentation for assaying corrosives and/or severe ocular irritants, using CAM of embryonated hen's egg [23]. The HET-CAM was described by Luepke to evaluate irritant/corrosive potential and allows the study of the immediate effects of administration of the test substance on membrane of embryonated hen's egg. This method is internationally validated [24].

Fresh fertile White Leghorn hen's eggs were obtained and candled prior to use to discard nonviable or defective eggs. Eggs were placed in an incubator at 37 ± 0.2°C and 58 ± 2% relative humidity for 8 days. The test compounds and controls were dissolved in 2.5% (w/v) solution of agarose to reach final concentrations of 25 *μ*g/*μ*L (250 *μ*g/pellet). For ease of application, pellets of these solutions were prepared by drop wise application of 10 *μ*L on parafilm and immediately cooled to room temperature for solidification [25]. On day 9, eggs were removed from the incubator; air cell of the eggs was marked, cut, and pared off without injuring the CAM. Pellets were placed directly onto the CAM and observed for 300 seconds for sign of hemorrhage or lysis reactions on the CAM. A 0.9% (w/v) sodium chloride (NaCl) and 1% (w/v) sodium dodecyl sulfate (SDS) in distilled water were used as negative control and positive control, respectively. The whole experiment was carried out under laminar airflow cabinet at room temperature.

After the application of the test substance, the chorioallantoic blood vessels and capillaries were examined for irritant effects. The irritant effects were hyperaemia, haemorrhage and clotting at different time points after application for 5 min [26]. A time-dependent numerical score was allocated to each test substance or formulation (Table 3). 

*Therapeutic Efficacy Studies in Rabbits.* The optimized NPs formulations were tested for their intraocular pressure lowering activity on normotensive albino rabbits (2–2.5 kg) and the results were compared to those of a marketed DRZ solution (2.0%). Various studies are reported citing normotensive rabbit as an appropriate animal model for therapeutic efficacy study [27]. The normotensive rabbit model was chosen due to its experimental feasibility and simplicity in interpretation of collected data. Institutional Animal Ethics Committee (IAEC) approved the pharmacological efficacy studies in rabbits. The animals were housed at controlled temperature (25 ± 2°C) and humidity (60 ± 5%), with a 12/12 h light-dark cycle. They had free access to food and water. The dispersions of sterile lyophilized NPs formulations in sterile distilled water were used in this study having a concentration of 2.0% w/v DRZ. The rabbits were divided in to three groups. Each group composed of three rabbits. Group 1, Group 2, and Group 3 received OCM-CSNPs, CSNPs, and marketed formulations, respectively. For measuring the IOP, cornea of each rabbit was anaesthetized by instilling 50 *μ*L xylocaine (2% w/v) solution before administration of formulation. All rabbits received a single 50 *μ*L dose of NPs and marketed formulation onto the corneal surface of rabbit's right eye. After instillation of formulation, the therapeutic efficacy of DRZ nanoparticles was assessed by measuring the IOP using a standardized tonometer (ShiØtz, Germany) [28]. IOP was measured after 30 min of drug administration and then every 1 hour over the period of 8 h. The left eye was left as a control in all the experimental animals. The ocular pressure lowering activity was expressed as average difference in IOP between the treated and control eye [29]. 

### 2.3. Statistical Analysis

Data are presented as the mean ± standard deviation. One-way analysis of variance (ANOVA) was performed followed by Dennett's multiple comparisons to test the statistical significance. The tests were performed by using GraphPad Prism version 6.01 for Windows, GraphPad Software (La Jolla CA, USA).

## 3. Results and Discussion

### 3.1. Synthesis of OCM-CS

Percentage yield of OCM-CS in IPA alone gave low yield (48.62%) which may be attributed to the fact that CS was not alkalized in the nonaqueous solvent. The use of water alone as solvent lowers the yield even more (5.68%) owing to the easy swelling of the previously formed OCM-CS in water to form jelly that coats the outside of the CS particle and inhibits the course of reaction. Therefore ratio of water/IPA was an important element for the successful completion of the reaction. The increase of the ratio of water/IPA in the reaction solvent decreases the fraction of carboxymethylation and increases the insolubility [15]. 

Similarly, when the effect of temperature on percent yield of OCM-CS was investigated, it was noted that the higher reaction temperature favoured the substitution of the carboxymethyl on the –OH group [14]. The highest yield 89.15% was obtained at 55°C (Table 2). Hence, 55°C temperature and solvent ratio H_2_O/IPA of 1/4 were considered optimum.

### 3.2. FT-IR Spectra of OCM-CS

**Figure 1:**
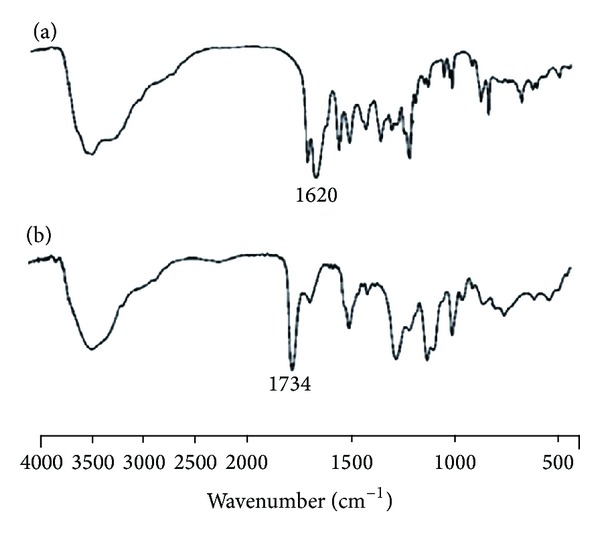
FT-IR spectrum of (a) CS and (b) OCM-CS. Intense peak at 1734 cm^−1^ in the IR spectrum of OCM-CS signifies the presence of –COOH group. Abbreviations: OCM-CS, *6-O*-carboxymethyl chitosan; CS, chitosan; FT-IR, Fourier transformation-infrared.

The basic characteristic peaks (Figure 1) of CS are at 3455 cm^−1^ (O–H stretch), 2923–2867 cm^−1^ (C–H stretch), 1598–1625 cm^−1^ (N–H bend), and 1094 cm^−1^ (C–O stretch). Compared with the peaks of CS, intense peak at 1734 cm^−1^ in the IR spectrum of OCM-CS signifies the presences of –COOH group. The 1076 cm^−1^ peak is attributable to C–O stretching of ether group of carboxymethyl moiety [30].

### 3.3. DSC Theromgram of OCM-CS

**Figure 2:**
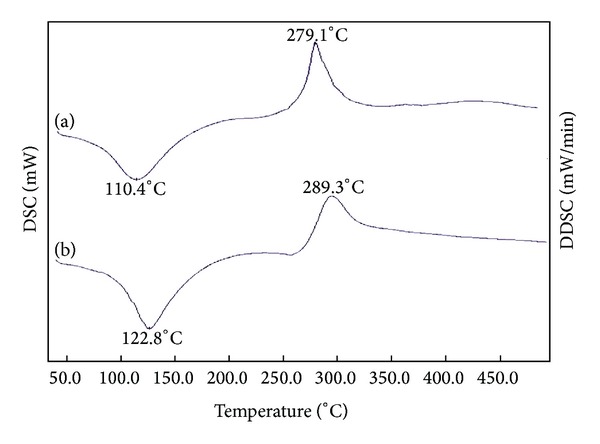
DSC thermograms of (a) CS and (b) OCM-CS. The endothermic and exothermic event for OCM-CS appeared at higher temperatures. Abbreviations: OCM-CS, *6-O*-carboxymethyl chitosan; CS, chitosan; DSC, differential scanning calorimetry.

The thermograms of CS and OCM-CS were characterized by two thermal events: the first endothermic and the second exothermic (Figure 2). The endothermic event appeared as a peak centered at 125–150°C. The exothermic event appeared as a peak centered at 270–330°C corresponding to the decomposition of the polymer. In contrast, both the peaks for CS appeared at lower temperatures (close to 100°C and 280°C, resp.) indicating the superior thermal stability of OCM-CS that was in accordance with the finding by Kittur et al., 2002 [31]. 

### 3.4. ^13^C NMR Spectroscopy

**Figure 3:**
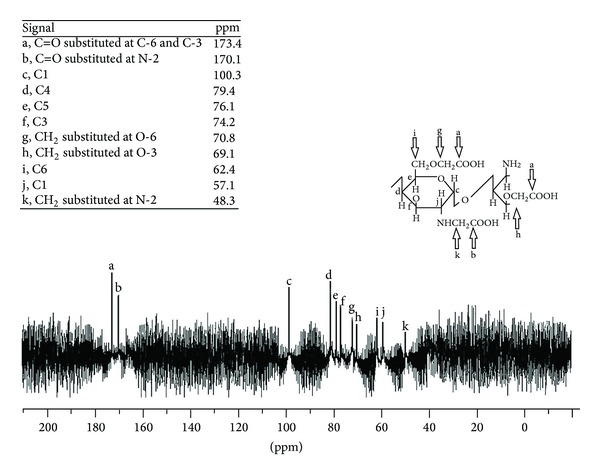
^13^C NMR spectrum of OCM-CS. Peak for –CH_2_ at O-6 is intense compare to that present at O-3 and N-2. Abbreviations: OCM-CS, *6-O*-carboxymethyl chitosan; C1–6, carbon atom of OCM-CS unit; C=O, carbonyl group; CH_2_, mehtylene group; O-3, O-6, oxygen present at the third and the sixth carbon of OCM-CS; N-2, nitrogen present at the second carbon atom of OCM-CS.

Evidence supporting the successful carboxymethylation of CS was provided by the ^13^C NMR spectrum of OCM-CS (Figure 3). The signals for –COOH substituted on –OH and –NH were present at 173.4 and 170.1 ppm, respectively. Chemical shifts at 70.9, 69.1, and 48.3 ppm were assigned to –CH_2_COOH groups substituted on O-6, O-3, and N-2, indicating that there were three possible sites for the carboxymethylation of CS. On account of the signal intensity, it was concluded that the OH-6 was the major site for carboxymethylation of CS [32, 33]. 

### 3.5. Content of Free Amino Group

**Figure 4:**
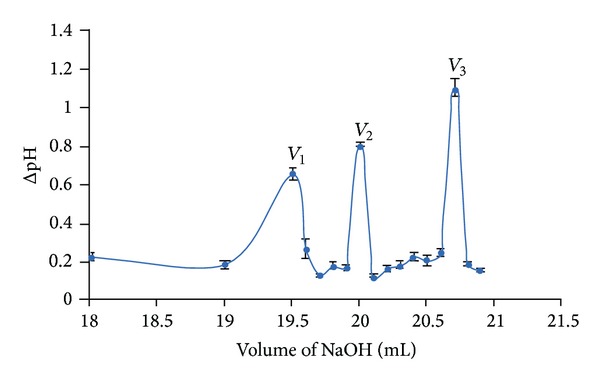
Graphical representation of potentiometric curve for content of free amino group. *V*_1_ and *V*_2_ represent the volume of 0.1 M NaOH needed to neutralize excess of free HCl and carboxyl group of OCM-CS. *V*_3_ is the volume of 0.1 M NaOH required to neutralize the carboxyl group and HCl associated with NH_2_ functional group of OCM-CS. Abbreviations: ΔpH, change in pH.

The content of free amino group was found to be 84.02%. *V*_1_ and *V*_2_ (Figure 4) represent the volume of 0.1 M NaOH needed to neutralize excess of free HCl and carboxyl group of OCM-CS, respectively. *V*_3_ is the volume of 0.1 M NaOH required to neutralize the carboxyl group and HCl associated with NH_2_ functional group of OCM-CS. Subtraction of *V*_2_ from *V*_3_ gives the volume of 0.1 M NaOH required to neutralize the HCl associated with the NH_2_ group of OCM-CS. From the result, it was clear that almost 16% of the amino groups (84.02% free amino group) present in the saccharide unit of OCM-CS molecule were also substituted with the carboxymethyl group during the synthesis of OCM-CS from CS.

### 3.6. Degree of Substitution

The value of DS was found to be 1.1576. It signified that, apart from –OH group at the sixth carbon (C_6_), some of the –OH group present at the third carbon (C_3_) and –NH_2_ groups present at the second carbon (C_2_) of the CS were also carboxymethylated which was confirmed from the content of amino group.

### 3.7. Preparation of OCM-CS and CS NPs

The weight ratios between OCM-CS : CaCl_2_ and CS : TPP are critical and controls the particle size and size distribution of the nanoparticles [19, 20]. The size characteristics have been found to affect the biological performance of NPs [34]. The changes in the PS and PI for series of OCM-CS : CaCl_2_ and CS : TPP weight ratios revealed that as the concentration of crosslinkers was increased, PS and PI of NPs were increased to NPs in micron range. The increase in CaCl_2_ and TPP concentration in the mixing ratio leads to agglomeration of OCM-CS and CS in NPs. The optimum OCM-CS : CaCl_2_ and CS : TPP weight ratios that resulted in particles of submicron range were found to be 4 : 1, 5 : 1, and 6 : 1. These ratios were loaded with 50% DRZ and NPs were studied for PS, PI, zeta potential, and entrapment efficiency.

### 3.8. Particle Size and PI of NPs

Particle size distribution of DRZ loaded OCM-CSNPs varied from 212.4 ± 0.79 nm to 500.4 ± 11.88 nm with PI varying from 0.244 ± 0.016 to 0.444 ± 0.028 as the weight ratio of OCM-CS : CaCl_2_ changed from 6 : 1 to 4 : 1. It was clear that by incrementing OCM-CS in the weight ratio, blank OCM-CSNPs of smaller sizes were produced (Table 4). Incorporation of the DRZ into OCM-CSNPs led to increase of their size compared with blank NPs. This could be attributed to reduction in ionic interactions between OCM-CS and CaCl_2_ during formation of NPs due to the positive charge induced on DRZ molecules by ionization in distilled water (pH 7) [20]. A similar trend was observed for CSNPs (Table 5). Particle size and PI varied from 250.3 ± 2.63 to 490.9 ± 4.80 and 0.442 ± 0.030 to 0.313 ± 0.009, respectively, as the CS : TPP weight ratio of DRZ loaded CSNPs was changed from 6 : 1 to 4 : 1.

### 3.9. Zeta Potential of NPs

Zeta potential values varied from −18.03 ± 0.404 to −28.57 ± 0.513 as the OCM-CS : CaCl_2_ weight ratio changed from 4 : 1 to 6 : 1. The negative zeta potential values for OCM-CSNPs were attributed to the presence of negatively charged carboxyl groups (COO^−^) [35]. When the proportion of OCM-CS in polymer: cross linker, weight ratio was high the zeta potential value was also high (Table 6). The high zeta potential value demonstrated the availability of excessive anionic charged on OCM-CSNPs. The zeta potential values in all ratios indicated the moderate stability of OCM-CSNPs. In the case of CSNPs, zeta potential values were positive, indicative of protonated amino group (NH_3_^+^) of CS. CSNPs followed the similar trend that with the increase of CS in CS : TPP weight ratio the positive zeta potential also increased indicating excess of NH_3_^+^ (Table 7).

### 3.10. Evaluation of Entrapment Efficiency of NPs

The entrapment efficiency of OCM-CSNPs for 50% DRZ loading was found to be 18.13 ± 0.47, 13.71 ± 0.53, and 9.37 ± 0.36 for OCM-CS : CaCl_2_ weight ratio 6 : 1, 5 : 1, and 4 : 1, respectively, (Table 6). OCM-CSNPs were formed due to ionic interactions between OCM-CS, CaCl_2_, and DRZ. As the concentration of OCM-CS increased from 4 : 1 to 6 : 1, entrapment efficiency also increased owing to availability of larger number of negatively charged COO^−^ group that interacted with positively charged NH_3_^+^ of DRZ. The amount of CaCl_2_ required for ionic gelation of OCM-CS containing DRZ was less than that required for plain OCM-CS solution. This indicates that the binding affinity of CaCl_2_ towards OCM-CS in presence of DRZ was reduced. 

For CSNPs, entrapment efficiency was found to be 20.08 ± 0.87, 16.29 ± 0.61, and 10.51 ± 0.57, respectively, for 6 : 1, 5 : 1, and 4 : 1 for CS : TPP weight ratio (Table 7). In this case, there was no ionic interaction (only weak electrostatic interaction) between CS and DRZ (both positively charged at pH 5.5) but negatively charged polyanionic TPP could interact with cationic CS and preferentially with protonated DRZ. Therefore, with increased concentration of TPP in CS : TPP weight ratio from 6 : 1 to 4 : 1, the amount of DRZ available for physical entrapment with CS decreased resulting in decreased entrapment. Similar results were obtained by Singh et al. for CSNPs of brimonidine tartrate which is also protonated at the same pH [36]. Therefore, CS : TPP weight ratio 6 : 1, with minimum amount of TPP resulted in the highest entrapment [20]. 

### 3.11. Effect of Drug Loading on Particle Size, Zeta Potential, and Entrapment Efficiency

**Figure 5:**
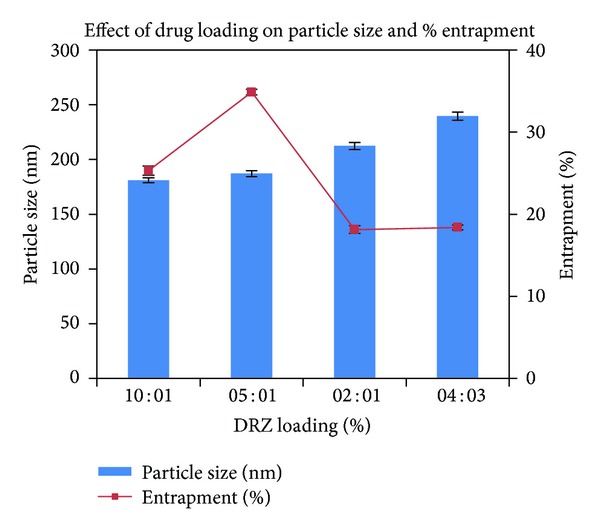
Effect of drug loading on OCM-CS : CaCl_2_ weight ratio of 6 : 1. Values are expressed as mean ± standard deviation, *n* = 3. Abbreviations: OCM-CS, *6-O*-carboxymethyl chitosan; CaCl_2_, calcium chloride; PS, particle size; EE, entrapment efficiency; DRZ, dorzolamide hydrochloride.

**Figure 6:**
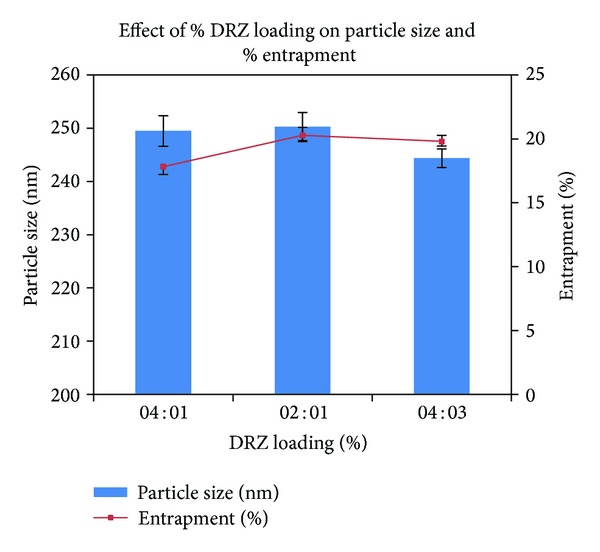
Effect of drug loading on CS : TPP weight ratio of 6 : 1. Values are expressed as mean ± standard deviation, *n* = 3. Abbreviations: CS, chitosan; TPP, tripolyphosphate; PS, particle size; EE, entrapment efficiency; DRZ, dorzolamide hydrochloride.

The OCM-CS : CaCl_2_ and CS : TPP weight ratios were loaded with a different polymer: DRZ and the effect on particle size, PI, zeta potential, and entrapment efficiency was studied (Table 8). For OCM-CSNPs, particle size increased with increased DRZ loading. The mean particle size varied from 181.1 ± 2.721 to 239.6 ± 3.811 nm with PI ranging from 0.209 ± 0.081 to 0.316 ± 0.029. The negative zeta potential values changed from −31.24 ± 0.75 to −24.03 ± 0.68 as the concentration of DRZ increased. These results demonstrated that the incorporation of the DRZ into OCM-CSNPs led to a drug proportion-dependent increase of their size compared with blank NPs (Figure 5). Increased drug proportion caused an increased reduction of OCM-CS : CaCl_2_ interaction, leading to increase NPs size [37], whereas no significant change in the particle size of CSNPs was observed as the drug loading was increased. The mean particle size was found to be in the range of 250.3 ± 2.627 nm with PI 0.313 ± 0.009 (Figure 6). Zeta potential values showed moderate to good stability (+28.61 ± 0.924 to +33.89 ± 0.547). These narrow particle size distributions with low PI and moderate zeta potential values attributed to stability of OCM-CS and CS NPs [38].

The entrapment efficiency for OCM-CSNPs was found to be 18.38 ± 0.29, 18.13 ± 0.47, 34.87 ± 0.33, and 25.29 ± 0.56 as the amount of DRZ decreased from 75% to 10% of DRZ loading (Table 8). DRZ exhibits two distinct p*K*_*a*_ values, 6.35 (p*K*_*a*1_) and 8.5 (p*K*_*a*2_) corresponding to the protonated secondary amino group and negatively charged sulfonamide group, respectively [39]. It exhibits, a cationic form at and below pH 6.4 and anionic form, at and above pH 8.5. The largest fraction of unionized form exists at pH right between the two p*K*_*a*_ values. The aqueous solubility of DRZ is a function of ionization constant (p*K*_*a*_) of the drug molecule. The pH solubility profile of DRZ exhibits lowest solubility between the two p*K*_*a*_ values. OCM-CS when dissolved in distilled water exhibited a pH of 7.4 ± 0.2. At this pH range, DRZ exhibited minimal solubility owing to its unionized form and the drug was protonated as the pH was below 8.5, increasing its soluble fraction [39]. When the pH of OCM-CS was lowered at and below 6.4, it resulted in precipitation of the polymer [15]. When the DRZ loading increased from 20–75%, the amount of DRZ entrapped decreased owing to its insolubility and unionized form. For this reason the entrapment efficiency of OCM-CSNPs at lower DRZ loading was higher. About 20% DRZ loaded NPs resulted in the highest entrapment and was selected as optimized. The hydrophilicity of DRZ poses difficulty in achieving high entrapment as it can easily come to the aqueous phase outside [40]. At higher DRZ concentration entrapment efficiency was reduced because the drug tends to precipitate. Considering all these factors, concentration of OCM-CS and DRZ was optimized so as to give better entrapment and desired size. The entrapment efficiency for CSNPs was found to be 17.83 ± 0.61, 20.28 ± 0.48, and 19.81 ± 0.37 as the amount of DRZ loading was increased from 25% to 75% (Table 9).

### 3.12. *In Vitro* Drug Release of NPs

**Figure 7:**
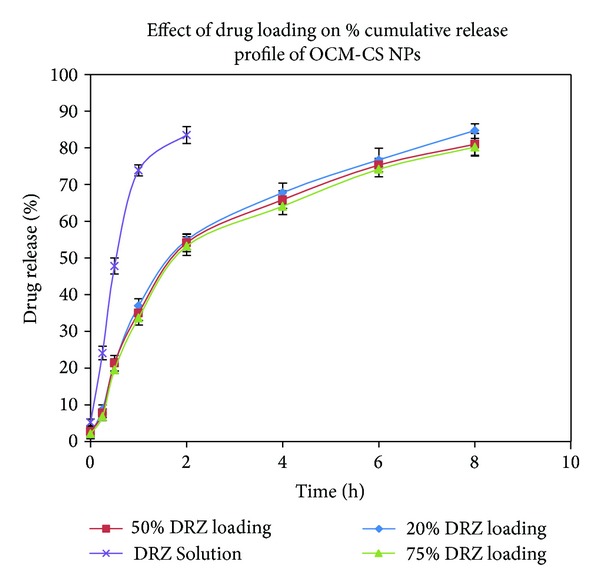
Drug release profile of OCM-CSNPs. Values are expressed as mean ± standard deviation, *n* = 3. Abbreviations: OCM-CSNPs, *6-O*-carboxymethyl chitosan nanoparticles; DRZ, dorzolamide hydrochloride.

**Figure 8:**
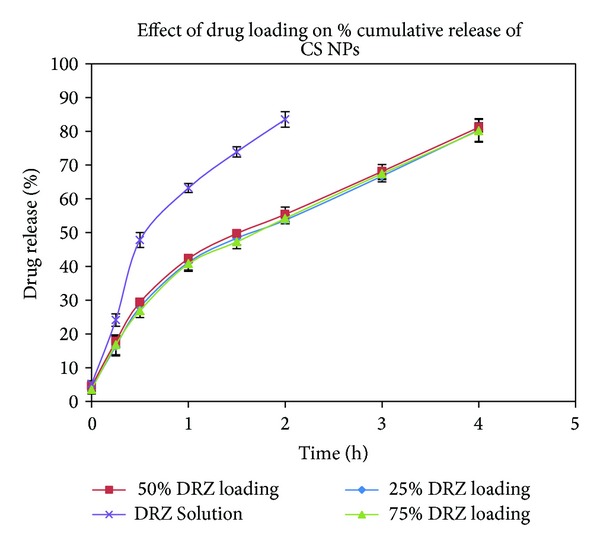
Drug release profile of CSNPs. Values are expressed as mean ± standard deviation, *n* = 3. Abbreviations: CSNPs, chitosan nanoparticles; DRZ, dorzolamide hydrochloride.

The *in vitro* release profiles of DRZ loaded OCM-CSNs were compared with those of DRZ from aqueous solution (Figure 7). The release profile for OCM-CSNPs followed a biphasic pattern, characterized by initial burst release followed by a prolonged release [19]. The burst release lasted for 60 min, releasing 30% to 35% drug. This initial burst release could be due to rapid dissolution of DRZ adsorbed on the surface of OCM-CSNs. After the initial burst release period, release rate was reduced and that could be due to diffusion of the drug through OCM-CSNs matrix. The release was sustained up to 8 h. CSNPs also followed biphasic release pattern with burst release of 30 min duration releasing 30% to 35% of drug and sustained release period of 4 h (Figure 8) [36]. 

### 3.13. FT-IR Spectroscopy of NPs

**Figure 9:**
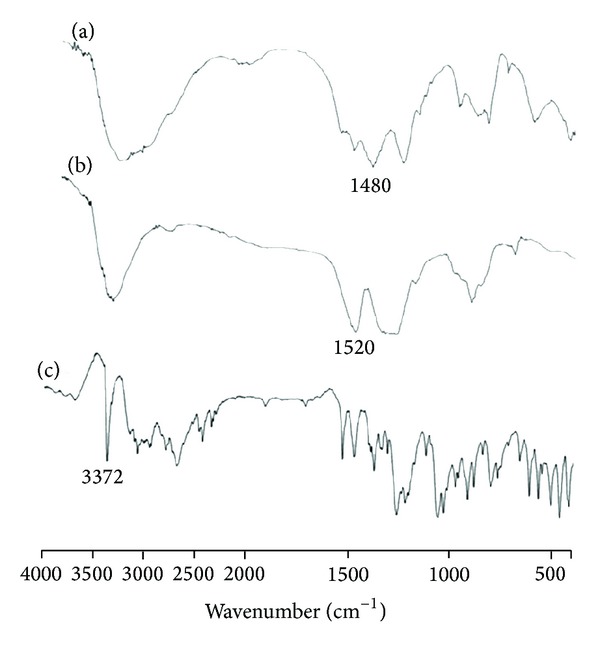
FT-IR spectra of (a) DRZ loaded OCM-CSNPs, (b) Blank OCM-CSNPs, and (c) DRZ powder. DRZ showed a strong absorbance at 3372 cm^−1^ attributed to the primary amino group. The same peak in OCM-CSNPs disappeared that was a clear indication and that the NH_2_^+^ of DRZ interacted strongly with –COOH of OCM-CS. Abbreviations: OCM-CSNPs, *6-O*-carboxymethyl chitosan nanoparticles; DRZ, dorzolamide hydrochloride; FT-IR, Fourier transformation-infrared.

In blank OCM-CSNPs, the peak at 1734 cm^−1^ shifted to lower values indicating an ionic interaction of –COOH with Ca^+2^ ion (Figure 9). This interaction reduced OCM-CS solubility and was responsible for OCM-CS separation from the solution in the form of NPs. When DRZ entrapped into the OCM-CSNPs, the peak at 1734 cm^−1^ shifted to lower values indicating an ionic interaction of –COOH with NH_2_^+^ of DRZ. DRZ had a strong absorbance at 3372 cm^−1^ attributed to the primary amino group. The same peak in OCM-CSNPs was disappeared and that was a clear indication that the NH_2_^+^ of DRZ interacted strongly with –COOH of OCM-CS.

**Figure 10:**
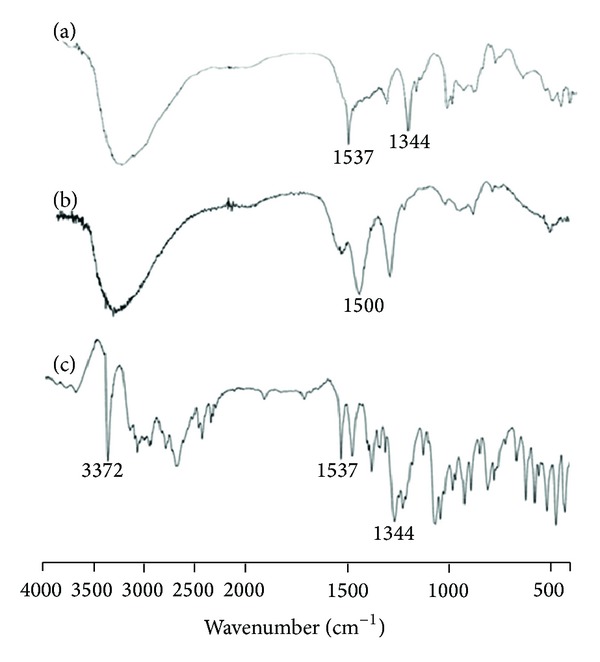
FT-IR spectra of (a) DRZ loaded CSNPs, (b) blank CSNPs, and (c) DRZ powder. The broadened peak in the range 3300–3400 cm^−1^ was due to overlap of the primary amino and hydroxyl peaks. The peaks of DRZ at 1537 and 1344 cm^−1^ were visible in DRZ loaded CSNs, a clear indication that no ionic interaction occurred between the DRZ and CS and the entrapment of DRZ was merely of a physical type. Abbreviations: CSNPs, chitosan nanoparticles; DRZ, dorzolamide hydrochloride; FT-IR, Fourier transformation infrared.

For CSNPs, no significant changes in the IR spectrum of the DRZ and DRZ loaded CSNPs occurred (Figure 10). The broadened peak in the range of 3300–3400 cm^−1^ was due to overlap of the primary amino and hydroxyl peaks. The peaks of DRZ at 1589, 1537, and 1344 cm^−1^ were visible in DRZ loaded CSNPs, a clear indication that no ionic interaction occurred between the DRZ and CS and the entrapment of DRZ was merely of a physical type [20]. 

### 3.14. DSC Analysis of NPs

**Figure 11:**
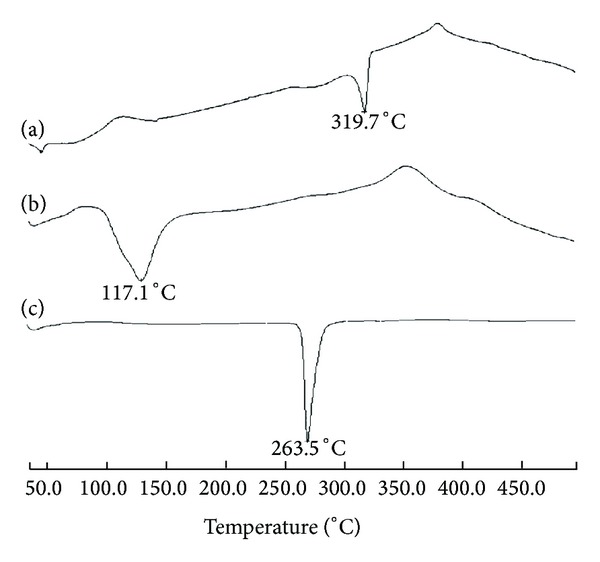
DSC thermograms of (a) DRZ loaded OCM-CSNPs, (b) Blank OCM-CSNPs, and (c) DRZ powder. Thermogram showed a shift in endotherm when DRZ was loaded showing a strong interaction of DRZ with OCM-CS. Abbreviations: OCM-CSNPs, *6-O*-carboxymethyl chitosan nanoparticles; DRZ, dorzolamide hydrochloride; DSC, differential scanning calorimetry.

**Figure 12:**
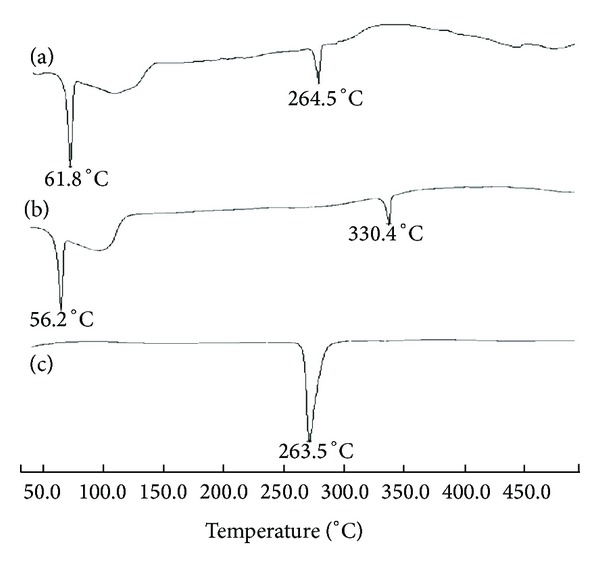
DSC thermograms of (a) DRZ loaded CSNPs, (b) Blank CSNPs and (c) DRZ powder. DRZ loaded CSNPs showed the prominent endotherm of DRZ indicating weak interaction of DRZ with CS. Abbreviations: CSNPs, chitosan nanoparticles; DRZ, dorzolamide hydrochloride; DSC, differential scanning calorimetry.

DSC data allow identification and characterization of a drug substance through the melting temperature and heat of fusion, in case of crystalline substances. Polymorphic forms can also be identified by DSC by virtue of their different melting temperature. Thermogram (Figure 11) for blank OCM-CSNPs showed a shift in endotherm value indicating interaction of OCM-CS with CaCl_2_. Also the thermogram showed a shift in endotherm when DRZ was loaded showing a strong interaction of DRZ with OCM-CS, whereas DRZ loaded CSNPs showed the prominent endotherm of DRZ indicating weak interaction of DRZ with CS (Figure 12).

### 3.15. *In Vitro* Mucoadhesion of NPs

OCM-CSNPs had higher mucin binding efficiency compared to CSNPs (Table 10). OCM-CS is an amphiprotic ether derivative which contains both the –COO^−^ and –NH_3_^+^ groups [30, 41]. OCM-CSNPs spontaneously adsorbed on the surface of the mucin, due to electrostatic attraction between the positively charged amino groups of OCM-CS and the negatively charged sialic acid group of mucin. Apart from ionic interaction, a strong hydrogen bonding was present due to hydrophilic carboxylic acid group [42, 43].

The blank OCM-CSNPs had the highest mucoadhesion. After drug loading, the mucoadhesive strength of OCM-CSNPs decreased owing to increased particle size of drug loaded NPs as compared to blank NPs [44]. An increase in NPs size would decrease the adsorption of mucin on NPs surface (specific surface area decreases with increase in particle size), leading to decreased mucoadhesive strength of NPs.

A similar trend was followed by CSNPs, with blank NPs being more mucoadhesive than the drug loaded NPs. The mucoadhesive strength of CSNPs was less compared to OCM-CSNs. 

### 3.16. Morphological Characterization of NPs

**Figure 13:**
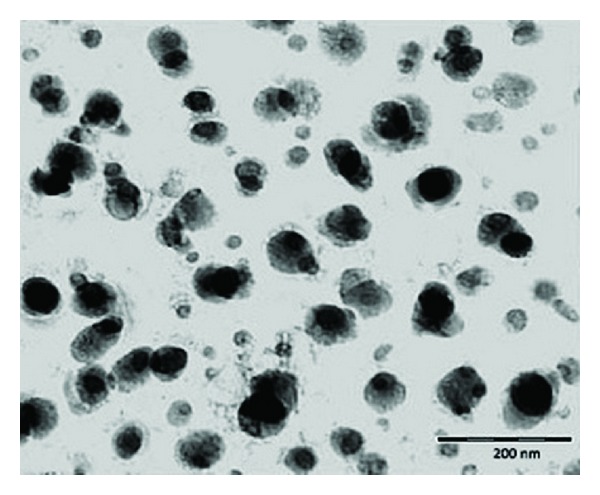
TEM photograph of DRZ loaded OCM-CSNPs. Bar 200 nm. Abbreviations: OCM-CSNPs, *6-O*-carboxymethyl chitosan nanoparticles; DRZ, dorzolamide hydrochloride; TEM, transmission electron microscopy.

TEM image of DRZ loaded OCM-CSNPs showed spherical shaped NPs (Figure 13). Discrete structure of the NPs could be attributed to negative surface charge. TEM image showed that the particle size ranged between 200 and 300 nm approximately which was in accordance with the particle size determined using DLS. 

### 3.17. Ocular Irritation Potential Test

**Figure 14:**
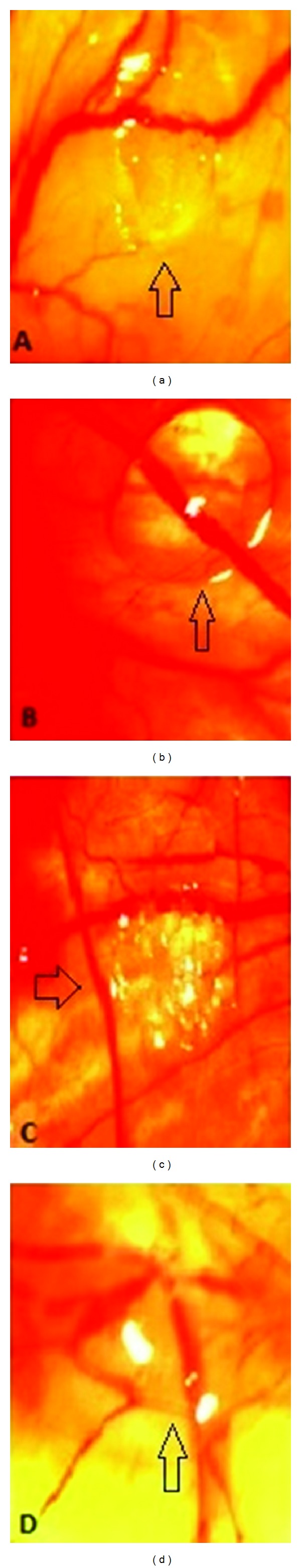
Vascular responses of control and test compound/formulations in the HET-CAM test (a) 0.9% NaCl, (b) 1% SDS, (c) 0.4% OCM-CS, and (d) OCM-CSNPs. Arrow mark in the figure indicate agarose pellet. Abbreviations: OCM-CS, *6-O*-carboxymethyl chitosan; OCM-CSNPs, *6-O*-carboxymethyl chitosan nanoparticles; NaCl, sodium chloride; SDS, sodium dodecyl sulphate; HET-CAM, hen's egg test chorioallantoic membrane.

Figure 14 outlines the effects of control and test substances after 5 min of pellet application on the CAMs. Only embryos having intact yolk and viable CAM were further incubated to day 9. Nine-day old CAMs were utilised for application of the pellets of control test substances. The temperature and relative humidity were kept at 37 ± 0.2°C and 58 ± 2% RH. These were found to be the optimum conditions for CAM growing [23].

**Figure 15:**
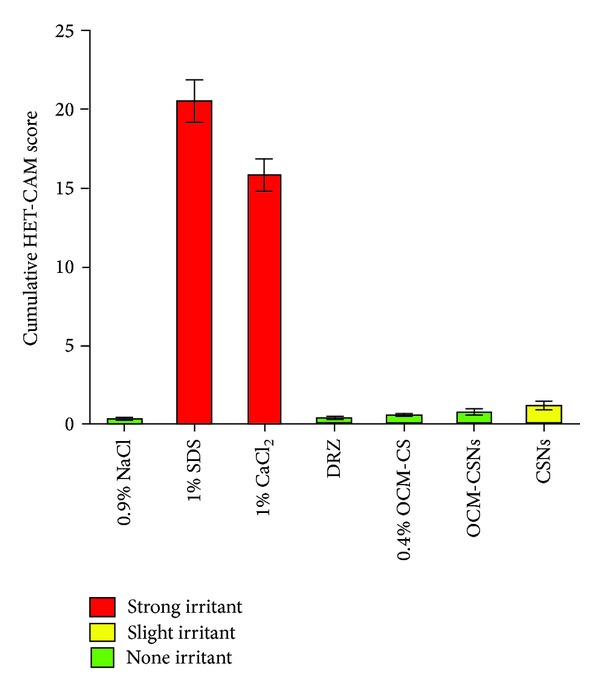
Cumulative HET-CAM scores of controls and test formulations. Values are expressed as mean ± standard deviation, *n* = 5. Abbreviations: HET-CAM, hen's egg test chorioallantoic membrane, NaCl, sodium chloride; SDS, sodium dodecyl sulphate; CaCl_2_, calcium chloride; DRZ, dorzolamide hydrochloride; OCM-CS, *6-O*-carboxymethyl chitosan; OCM-CSNPs, *6-O*-carboxymethyl chitosan nanoparticles; CSNPs, chitosan nanoparticles.

Figure 15 shows the cumulative HET-CAM scores for the controls, prepared NPs formulations. The average cumulative scores calculated for OCM-CSNs, 0.4% w/v OCM-CS and 0.9% NaCl were found to be <0.9. These results revealed that these test substances are practically nonirritant when applied to the surface of the CAM. In contrast, CSNPs were slightly irritant with a cumulative score of 1.2 ± 0.25, which could be attributed to acidic pH. A solution of 1% w/v CaCl_2_ was found to be strongly irritant whereas OCM-CS NPs showed no irritation. It is likely that the amount of CaCl_2_ in OCM-CSNPs was insufficient to produce an irritant effect. Another possible explanation could be that CaCl_2_ molecules are involved in interaction and bound to polymer and not present in free form, which is likely to reduce their interaction with the ocular surface.

### 3.18. Therapeutic Efficacy Studies in Rabbits

**Figure 16:**
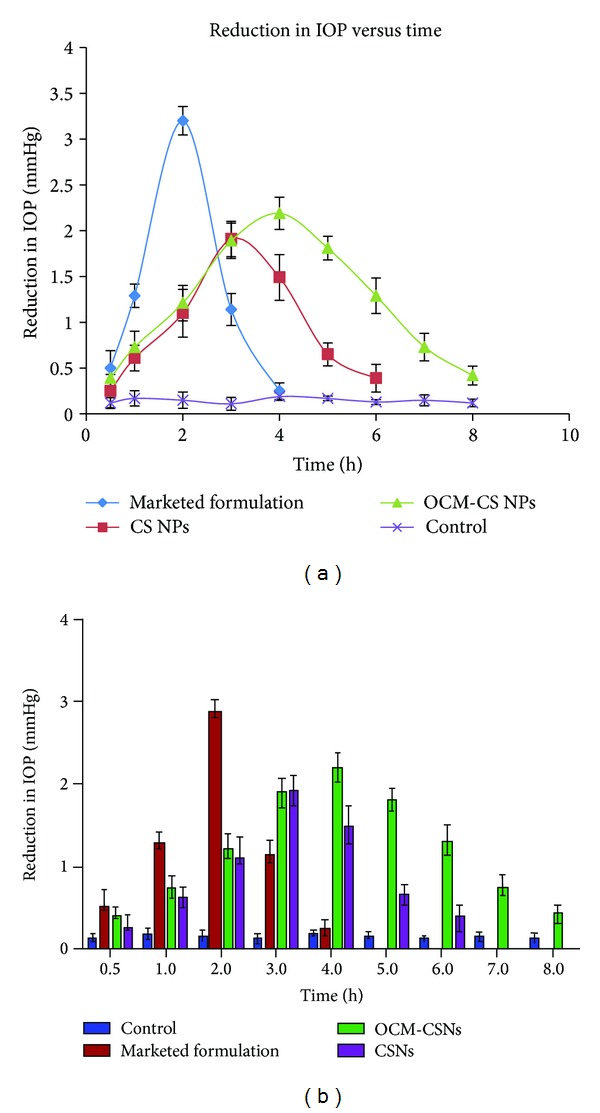
(a) Comparative therapeutic efficacy study of the DRZ loaded OCM-CSNPs, CSNPs, marketed formulation and control. (b) Application of ANOVA to efficacy data. Values are expressed as mean ± standard deviation, *n* = 3. Abbreviations: DRZ, dorzolamide hydrochloride; OCM-CSNPs, *6-O*-carboxymethyl chitosan nanoparticles; CSNPs, chitosan nanoparticles; ANOVA, analysis of variance.

The values of the reduction in IOP (mm of Hg) in normotensive albino rabbits after instillation of a 50 *μ*L dose of each NPs formulation as a function of time were compared to marketed formulation [45]. It was observed that the IOP lowering activity of marketed formulation reached to maximum value of 2.87 mm of Hg within 2 hr after instillation. This effect markedly decreased and abolished completely within 4 h whereas NPs formulation produced a significant sustained reduction in IOP. DRZ loaded OCM-CSNPs showed pharmacological effect that was sustained up to 8 h. The peak effect was observed at the 4th hour with reduction of IOP value by 2.19 mm of Hg, which was less than marketed formulation owing to slow release of drug from NPs compared to marketed formulation, whereas DRZ loaded CSNPs showed pharmacological effect, which was sustained up to 6 h. The peak effect was observed at the third hour with reduction of IOP value by 1.91 mm of Hg. As shown in Figure 16, developed OCM-CSNPs and CSNPs formulations showed statistically significant response when compared to the control group. Marketed formulation being solution showed pulse effect due to immediate availability of drug in large concentration. In case of NPs, drug was embedded/crosslinked in polymer matrix; large concentration of drug was not available immediately to produce the pulse effect. The prolonged duration of action was due to increased mucoadhesion of OCM-CS that interact with mucin effectively compared to CS. The mucoadhesion phenomenon is independent of tear turnover rate and depends on the mucus turnover rate that is generally more than 15 h. 

Hence, developed formulation of DRZ loaded OCM-CSNPs and CSNPs was found to be effective in lowering the IOP of eye when compared to marketed formulation. Thus, OCM-CSNPs showed better efficacy than CSNPs, which was attributed to better mucoadhesion of OCM-CS. 

## 4. Conclusion

In this study, OCM-CS was successfully synthesized from CS and characterized. DRZ loaded NPs were fabricated using OCMCS : CaCl_2_ and CS : TPP weight ratio of 6 : 1, respectively, by ionic gelation technique. 20% and 50% DRZ loading showed the highest entrapment efficiency for OCM-CSNs and CSNs, respectively. Entrapment efficiency of DRZ with synthesized OCM-CS improved by 14% compared to CS. *In vitro* release profile showed sustained release over the period of 8 h for OCM-CSNP. *In vitro* mucoadhesion studies showed enhanced mucoadhesion of OCM-CSNPs compared to CSNPs. Prepared OCM-CSNPs were nonirritant when tested by HET-CAM. *In vivo* studies of DRZ loaded OCM-CSNPs exhibited a promising prolonged antiglaucoma effect without pulse entry as compared to CSNPs. The resultant OCM-CSNPs had better entrapment, tailored drug release, and improved bioavailability with reduction in pulse entry as compared to CSNPs. Hence it can be concluded that DRZ loaded OCM-CSNPs is better and efficacious alternative to conventional eye drops for the anti-glaucoma activity. 

## Figures and Tables

**Table 1:** Effect of solvent ratios on yield of OCM-CS.

Serial number	Samples	H_2_O/IPA	Yield (%)
1	OCM-CS-1	1/9	75.39 ± 1.15
2	OCM-CS-2	0/10	48.62 ± 0.89
3	OCM-CS-3	2/8 (1/4)	89.13 ± 2.14
4	OCM-CS-4	5/5 (1/1)	78.54 ± 1.53
5	OCM-CS-5	8/2 (4/1)	16.29 ± 0.92
6	OCM-CS-6	10/0	5.68 ± 0.45

Values are expressed as mean ± standard deviation, *n* = 3.

OCM-CS: *6*-*O*-carboxymethyl chitosan; IPA: isopropyl alcohol; H_2_O: water.

**Table 2:** Effect of reaction temperature on yield of OCM-CS.

Serial number	Samples	Temperature (°C)	Yield (%)
1	OCM-CS-1	5	7.89 ± 0.85
2	OCM-CS-2	25	65.21 ± 1.87
3	OCM-CS-3	35	70.59 ± 1.58
4	OCM-CS-4	45	73.87 ± 1.98
5	OCM-CS-5	55	89.15 ± 3.25

Values are expressed as mean ± standard deviation, *n* = 3.

OCM-CS: *6*-*O*-carboxymethyl chitosan.

**Table 3:** Irritation scores and interpretations used in HET-CAM test.

Effect/time (min)	Score			Cumulative score	Irritation assessment
	0.5	2	5	0–0.9	None
Hyperemia	5	3	1	1.0–4.9	Slight
Hemorrhage	7	5	3	5.0–8.9	Moderate
Clotting/coagulation	9	7	5	9.0–21.0	Severe

**Table 4:** Effect of drug loading on PS and PI of OCM-CSNs.

OCMCS : CaCl_2_ weight ratio	PS of drug loaded NPs (nm)	PI	PS of blank NPs (nm)
6 : 1	212.4 ± 0.79	0.244 ± 0.016	174.0 ± 1.29
5 : 1	325.1 ± 5.31	0.384 ± 0.008	296.0 ± 6.85
4 : 1	500.4 ± 11.88	0.444 ± 0.028	481.8 ± 14.88

Values are expressed as mean ± standard deviation, *n* = 3.

OCM-CS: *6*-*O*-carboxymethyl chitosan; CaCl_2_: calcium chloride; PS: particle size; NPs: nanoparticles; PI: polydispersity index; OCM-CSNs: *6*-*O*-carboxymethyl chitosan nanoparticles.

**Table 5:** Effect of drug loading on PS and PI of CSNs.

CS : TPP weight ratio	PS of drug loaded NPs (nm)	PI	PS of blank NPs (nm)
6 : 1	250.3 ± 2.627	0.313 ± 0.009	247.5 ± 3.379
5 : 1	383.2 ± 3.668	0.385 ± 0.006	355.3 ± 6.102
4 : 1	490.9 ± 4.804	0.442 ± 0.030	446.7 ± 9.628

Values are expressed as mean ± standard deviation, *n* = 3.

CS: chitosan; TPP: tripolyphosphate; PS: particle size; NPs: nanoparticles; PI: polydispersity index; CSNs: chitosan nanoparticles.

**Table 6:** Effect of drug loading on zeta potential and EE of OCM-CSNs.

OCM-CS : CaCl_2_ weight ratio	Zeta potential (mV)		EE of OCM-CSNs
	DRZ loading (50%)	Blank OCM-CSNs	
6 : 1	−28.57 ± 0.513	−32.57 ± 0.571	18.13 ± 0.47
5 : 1	−24.57 ± 1.858	−27.19 ± 0.337	13.71 ± 0.53
4 : 1	−18.03 ± 0.404	−24.87 ± 0.259	9.37 ± 0.36

Values are expressed as mean ± standard deviation, *n* = 3.

OCM-CS: *6*-*O*-carboxymethyl chitosan; CaCl_2_: calcium chloride; NPs: nanoparticles; EE: entrapment efficiency; OCM-CSNs: 6-*O*-carboxymethyl chitosan nanoparticles.

**Table 7:** Effect of drug loading on zeta potential and EE of CSNs.

CS : TPP weight ratio	Zeta potential (mV)		EE of CSNs
	DRZ loading (50%)	Blank CSNs	
6 : 1	+32.47 ± 0.723	+28.58 ± 0.421	20.08 ± 0.87
5 : 1	+25.33 ± 0.404	+23.79 ± 0.234	16.29 ± 0.61
4 : 1	+22.13 ± 0.351	+21.58 ± 0.347	10.51 ± 0.57

Values are expressed as mean ± standard deviation, *n* = 3.

CS: chitosan; TPP: tripolyphosphate; EE: entrapment efficiency; NPs: nanoparticles; CSNs: chitosan nanoparticles.

**Table 8:** Effect of drug loading on OCM-CS : CaCl_2_ weight ratio of 6 : 1.

DRZ loading (%)	PS (nm)	PI	Zeta potential (mV)	EE of OCM-CSNs
Blank OCM-CSNs	174.0 ± 1.29	0.269 ± 0.021	−32.57 ± 0.57	—
10%	181.1 ± 2.19	0.209 ± 0.081	−31.24 ± 0.75	25.29 ± 0.56
20%	187.1 ± 2.72	0.219 ± 0.006	−30.87 ± 0.86	34.87 ± 0.33
50%	212.4 ± 3.21	0.244 ± 0.016	−28.57 ± 0.51	18.13 ± 0.47
75%	239.6 ± 3.81	0.316 ± 0.029	−24.03 ± 0.68	18.38 ± 0.29

Values are expressed as mean ± standard deviation, *n* = 3.

OCM-CS: *6*-*O*-carboxymethyl chitosan; CaCl_2_: calcium chloride; PS: particle size; NPs: nanoparticles; PI: polydispersity index; EE: entrapment efficiency; OCM-CSNs: *6*-*O*-carboxymethyl chitosan nanoparticles; DRZ: dorzolamide hydrochloride.

**Table 9:** Effect of drug loading on CS : TPP weight ratio of 6 : 1.

DRZ loading (%)	PS (nm)	PI	Zeta potential (mV)	EE of CSNs
Blank CSNs	247.5 ± 3.379	0.259 ± 0.009	27.58 ± 0.42	—
25%	249.5 ± 3.866	0.267 ± 0.005	+28.61 ± 0.92	17.83 ± 0.61
50%	250.3 ± 2.627	0.313 ± 0.009	+33.47 ± 0.72	20.28 ± 0.48
75%	244.4 ± 1.735	0.323 ± 0.040	+33.89 ± 0.55	19.81 ± 0.37

Values are expressed as mean ± standard deviation, *n* = 3.

CS: chitosan; TPP: tripolyphosphate; PS: particle size; NPs: nanoparticles; PI: polydispersity index; EE: entrapment efficiency; CSNs: chitosan nanoparticles; DRZ: dorzolamide hydrochloride.

**Table 10:** Mucin binding efficiency of NPs.

Sr. no.	Formulation	Mucin binding efficiency (%)
1	OCM-CSNs (blank)	49.6 ± 0.68
2	CSNs (blank)	41.6 ± 0.87
3	DRZ loaded OCM-CSNs	37.76 ± 0.92
4	DRZ loaded CSNs	28.16 ± 0.46

Values are expressed as mean ± standard deviation, *n* = 3.

NPs: nanoparticles; OCM-CSNs: *6*-*O*-carboxymethyl chitosan nanoparticles; CSNs: chitosan nanoparticles; DRZ: dorzolamide hydrochloride.
